# Regulatory T Cells Promote Overexpression of Lgr5 on Gastric Cancer Cells via TGF-beta1 and Confer Poor Prognosis in Gastric Cancer

**DOI:** 10.3389/fimmu.2019.01741

**Published:** 2019-07-30

**Authors:** Xiao-Sun Liu, Xian-Ke Lin, Ying Mei, Sabir Ahmad, Chong-Xian Yan, Hai-Long Jin, Hang Yu, Chao Chen, Cai-Zhao Lin, Ji-Ren Yu

**Affiliations:** ^1^Department of Gastrointestinal Surgery, The First Affiliated Hospital, Zhejiang University School of Medicine, Hangzhou, China; ^2^Key Laboratory of Precision Diagnosis and Treatment for Hepatobiliary and Pancreatic Tumor of Zhejiang Province, Hangzhou, China; ^3^Department of Colorectal Surgery, The First Affiliated Hospital, Zhejiang University School of Medicine, Hangzhou, China

**Keywords:** LGR5 protein, human, stomach neoplasms, T-lymphocytes, regulatory, transforming growth factor beta1, immune microenviroment

## Abstract

**Background:** The leucine-rich repeat containing G protein-coupled receptor 5 (Lgr5) is considered a cancer stem cell marker, and is often overexpressed in tumors. The interaction between Lgr5 and the immune-related tumor microenvironment is not completely understood. The aim of this study was to examine the role of Lgr5 in the microenvironment of gastric cancer (GC), and to explore possible immunological mechanisms influencing Lgr5 expression that are governed by regulatory T cells.

**Methods:** Lgr5 expression was examined in 180 GC tumors by immunohistochemistry, and in 80 pairs of GC tumors for analysis of Th1/Th2 cytokines by ELISA. In addition, SGC7901 cells were co-cultured with patient-derived Tregs, varying concentrations of TGF-β1, TGF-β1 neutralizing antibody, or TGF-β receptor inhibitor SB431542, and Lgr5 and β-catenin expression were examined by qRT-PCR and western blot.

**Results:** In this study, an immunosuppressive microenvironment was associated with high Lgr5 expression in GC. Furthermore, Lgr5 expression was up-regulated in GC cells co-cultured with Tregs or treated with exogenous TGF-β1. This up-regulation was partially inhibited by the TGF-β1 neutralizing antibody, or TGF-β1 receptor antagonist SB431542. β-catenin was up-regulated with high Lgr5 expression induced by exogenous TGF-β1, and this up-regulation was inhibited by SB431542. An increased number of Tregs and high Lgr5 expression in GC tissues were significantly associated with low overall survival.

**Conclusion:** Tregs promoted increased Lgr5 expression in GC cells via TGF-β1 and TGF-β1 signaling pathway, which may involve activation of the Wnt signaling pathway. High Lgr5 expression via TGF-β confer poor prognosis in gastric cancer.

## Introduction

Leucine-rich repeat containing G protein-coupled receptor 5 (Lgr5) is a global stem cell marker and a downstream target of Wnt/β-catenin signaling (1). Lgr5 is overexpressed in gastrointestinal tumors and other types of cancer, compared to its relatively low expression in normal tissues (2–5). In recent years, the role of Lgr5 in human gastrointestinal tumor development and progression has been increasingly studied (6–8). Indeed, systematic reviews and meta-analyses examining the significance of Lgr5 expression in tumors have shown that Lgr5 is a predictive factor for tumor invasion and metastasis, as well as an indicator of poor prognosis in gastric cancer (GC) and colorectal cancer (CRC) (2, 6). Furthermore, it has been reported that Lgr5 overexpression is associated with resistance to chemotherapy in these cancer types (7, 9, 10). In addition, several studies have suggested that Lgr5 might be a potential target for anti-tumor therapy in GC and CRC (11, 12). Indeed, targeting LGR5^+^ cells with an antibody-drug conjugate has been shown to be safe and have potent tumor efficacy in CRC (12). An immunosuppressive tumor microenvironment has been associated with a poor outcome in GC patients, mainly due to its permissive role in the facilitation of tumor invasion and metastasis by inhibiting anti-tumor defense mechanisms (13, 14). Many different immune cell types and cytokines have been reported to play a role in the immunosuppressive tumor microenvironment, as well as in the escape of tumor cells (15, 16). Nevertheless, little is known about the specific interactions between Lgr5 and the immunosuppressive tumor microenvironment in gastrointestinal tumors.

Regulatory T cells (Tregs), are immune cells that are responsible for the maintenance of immunological tolerance to self-antigens, as well as for the suppression of excessive immune responses, which would otherwise have deleterious consequences. However, it has been shown that they are also involved in immune evasion by tumor cells through the suppression of effector T cells and secretion of various cytokines (16). It has been established that transforming growth factor beta 1 (TGF-β1), interleukin (IL)-10, and IL-35 are key mediators of negative immune regulation by Tregs (17). Tregs have the ability to suppress the activity of other lymphoid cells, and play a central role in the inhibition of anti-tumor immunity in GC (18). Previous studies have shown that the number of Tregs is increased in tumor tissues, perigastric lymph nodes, and the peripheral blood of GC patients and in GC animal models (19, 20). In addition, it has been shown that depletion of Tregs, or blockade of inhibitory cytokines, can enhance the anti-tumor immunity, thereby improving the prognosis of gastrointestinal tumors in animal models (21). Indeed, our group and other groups have demonstrated that Tregs and TGF-β1 play important roles in creating a human GC immunosuppressive microenvironment (22–24). In addition, a high number of intratumoral Tregs significantly correlates with poor prognosis in GC (19).

TGF-β1, as one of the most pleiotropic cytokines, has different immune modulating functions in normal tissues and in tumor tissues (25). In normal gastrointestinal tissue, TGF-β1 acts as a tumor suppressor. However, in gastrointestinal cancers, tumor cells resist TGF-β1 growth suppression. In fact, TGF-β1 actually promotes cancer progression by mediating immune suppression, angiogenesis, and epithelial-to-mesenchymal transition (EMT) (25). In addition, previous studies have demonstrated that TGF-β1 acts as a tumor promoter in advanced colorectal cancer (26). Moreover, it has been shown that disruption of both TGF-β1 and Wnt signaling pathways synergistically drives CRC tumorigenesis and progression *in vivo* (27, 28). In intestinal organoid cultures and mouse models, an initiating mutation in Lgr5^+^ CRC stem cells occurs in the β-catenin or adenomatous polyposis coli (APC) gene, which then induces the activation of the Wnt signaling pathway (29). In addition, other *in vitro* and *in vivo* studies have demonstrated that Lgr5 expression is critical for the promotion of gastrointestinal cancer cell proliferation via β-catenin signaling (30).

Collectively, these studies suggest that Lgr5 may play an important role in the Treg- and TGF-β1-mediated cancer immunosuppressive microenvironment, although the exact immunological mechanism is still unknown. In this study, we aimed to examine the interactions between Lgr5 and the Treg-mediated tumor immunosuppressive microenvironment in human GC, as well as mechanisms by which Tregs promote Lgr5 overexpression in GC.

## Materials and Methods

### Ethics Statement

This study was approved by the ethical committee of the First Affiliated Hospital, Medical College, Zhejiang University (Hangzhou; Ethical number: 2017380). Written informed consent was obtained from all participants included in the study.

### Patients

All patients included in the study were recruited from the First Affiliated Hospital, Medical College, Zhejiang University (Hangzhou). Patients who met the following criteria were selected: a) diagnosis of gastric adenocarcinoma based on pathology, and b) effective surgical resection (according to the 7th edition of the American Joint Committee on Cancer). Patients were excluded from this study on the following conditions: (a) evidence of distant metastasis, therefore inoperable, (b) evidence of concurrent autoimmune disease, or (c) received anticancer therapy before surgery.

Formalin-fixed and paraffin-embedded (FFPE) GC tissues were collected from 100 patients from February 2009 to March 2010 for immunohistochemistry (IHC) and immunofluorescence (IF). In addition, 80 GC patients were recruited from May 2013 to May 2014, and their tumors as well as corresponding normal mucosal tissue were obtained during surgery and stored at −80°C until further use for ELISA analysis. Corresponding FFPE tissue taken from these patients was used for IHC. Twenty GC patients recruited from May 2013 to May 2014 had peripheral blood samples collected for Tregs isolation and use. Clinicopathological data were also recorded, including patient age and sex, histological type of tumor, evidence of lymphovascular invasion, and the tumor/lymph node/metastasis (TNM) pathological stage according to the 7th edition of the American Joint Committee on Cancer.

### IHC and IF

IHC and IF analyses were performed as described in our previous study (31). For IHC analysis, primary antibodies included rabbit anti-Lgr5 (1:800; Abcam-ab75732, USA), and mouse anti-FoxP3 (1:400; Abcam-ab20034, USA). Secondary antibodies used for this analysis were peroxidase-conjugated goat anti-mouse/anti-rabbit secondary antibodies (859073, Invitrogen, USA). For IF analysis, the same primary antibodies were used, while the secondary antibodies included donkey anti-mouse (Alexa Fluor 488, 1:200, Invitrogen, USA) and donkey anti-rabbit (Alexa Fluor 568, 1:200, Invitrogen, USA).

### Quantification of IHC Parameters

FoxP3^+^ Treg data were obtained by manually counting positively stained cells in 10 random fields of both normal and intratumoral regions under 400^×^ ocular lens, microscopic high power field (HPF; Olympus). Random high power fields were selected by two independent, blinded observers (YM and XKL), and count needed to be redone if there was a statistical difference between the means of the two groups. Lgr5 expression was scored according to the intensity of the dye color, and the percentage of positive cells in the tumor tissue. The intensity of the dye was graded as 0 (no color), 1 (light yellow), 2 (light brown), or 3 (brown), and the percentage of positive cells was graded as 0 (<5%), 1 (5–25%), 2 (25–50%), 3 (51–75%), or 4 (>75%) (32). These two grades were multiplied and specimens were assigned to four groups according to the achieved score: 0–3 (negative), 4–6 (weak positive), 7–9 (positive), and 10–12 (strong positive).

### ELISA

Fresh frozen human GC tissues and corresponding normal mucous tissues were used for the detection of Th1/Th2 cytokines by ELISA as described previously (22). For this purpose, human IL-2, IL-4, IL-5, IL-6, IL-10, interferon gamma (IFN-γ), tumor necrosis factor alpha (TNF-α) and TGF-β1 (eBioscience, USA) ELISA kits were utilized.

### Cell Isolation and Sorting

Peripheral blood mononuclear cells (PBMCs) were obtained from the peripheral blood of GC patients and used for magnetic-activated cell sorting (MACS) using the human CD4^+^CD25^+^CD127^dim/−^ Regulatory T Cell Isolation Kit II (Miltenyi Biotec, Germany).

### Cell Culture and Treatment

Human GC cell lines (AGS, SGC-7901, MGC-803, MKN45, BGC-823 and KATO III) were obtained from the Chinese Academy of Medical Sciences. Purified Tregs were co-cultured with SGC7901 cells in RPMI-1640 supplemented with penicillin (100 U/mL), streptomycin (100 mg/mL), L-glutamine (2 mM), beta-hydroxy ethyl alcohol (55 mM, Gibco, Australia), sodium pyruvate (10 mM, Sigma, USA) and 10% FBS (Gibco, Australia). Next, SGC7901 cells were respectively, treated with 1 × 10^4^ purified Tregs or various concentrations of recombinant TGF-β1 protein (R&D Systems, 240-B-002 USA), with or without human TGF-β1 neutralizing antibody (AF-246-NA, R&D Systems, USA) or the TGF-β/ALK5/Smad2 inhibitor (SB431542, Abcam, USA). Lgr5 and β-catenin expression were examined by qRT-PCR and western blot.

### Flow Cytometry Analysis

Membrane Lgr5 staining in SGC7901 cells was performed using mouse anti-Lgr5 PE-conjugated antibody (FAB8078P, R&D, USA) and the isotype control was a mouse monoclonal IgG PE-conjugated antibody (IC003P, R&D, USA). The purity range of isolated CD4^+^CD25^+^CD127^dim/−^ Tregs was measured by flow cytometry with the Human Regulatory T Cell Staining Kit (eBioscience, USA) and human CD25-APC antibody (130-109-076. Miltenyi Biotec, Germany).

### Quantitative RT-PCR

Primers used for Lgr5 and β-catenin mRNA expression analysis were obtained from Sangon Biotech (China) and their sequences were as follows: 5′-GTCCAACCTCCTGTCGTCTT-3′ and 5′-GGCATTCTCACACACTCCAA-3′ for Lgr5 and, 5′-CGACACCAAGAAGCAGAGAT-3′ and 5′-CGAATCAATCCAACAGTAGCC-3′ for β-catenin. Glyceraldehyde 3-phosphate dehydrogenase (GAPDH, 5′-CAGGAGGCATTGCTGATGAT-3′ and 5′-GAAGGCTGGGGCTCATTT-3′) was used as an endogenous control. For analysis, Lgr5 and β-catenin expression were normalized to GAPDH expression.

### Western Blot Analysis

For western blot analysis, primary antibodies included rabbit anti-Lgr5 (1:1,000, Abcam- ab75732, USA) and rabbit anti-β-catenin antibody (1:5,000, Abcam- ab32572, USA). Secondary antibodies used for this analysis were respectively horse radish peroxidase (HRP)-conjugated goat anti-rabbit (GAR007, Dawenbiotec, China) and goat anti-mouse (WD0990, Dawenbiotec, China). β-actin (1:1000; Sigma-Aldrich, USA) was used as the loading control.

### Data Analysis

An unpaired t-test was used for single comparisons of groups with equal variance and normal distribution. Correlations between the expression of different cytokines and intratumor FoxP3^+^ Tregs were assessed using Pearson's or Spearman's correlation coefficient. Chi-squared test was used to assess the relationship between intratumor FoxP3^+^ Tregs, Lgr5 expression, and clinicopathological features of patients and their tumors. Overall survival (OS) was defined as the interval between the date of surgery and date of death or last follow-up, whichever occurred earlier. Survival function estimates were computed using the Kaplan-Meier method. A Cox proportional hazards model was used to assess the effect of FoxP3^+^ Tregs and Lgr5 expression in tumor tissues on overall survival. A two-sided *P*-value < 0.05 was considered to indicate statistical significance.

## Results

### High Lgr5 Expression in GC Tumors Was Associated With Poor Prognosis

**Figure 1:**
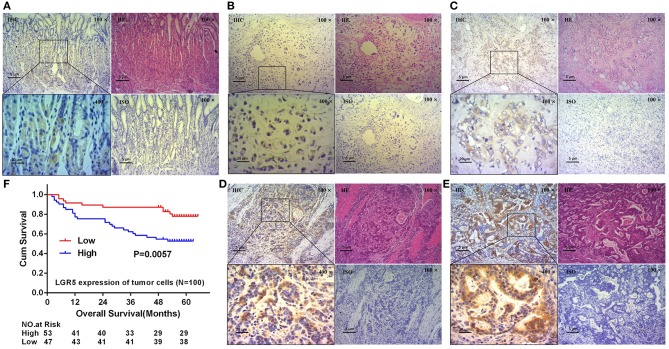
Expression of Lgr5 on normal gastric glands and GC tissues, and its prognostic significance. H&E and IHC staining on normal gastric glands and longitudinal sections shown that Lgr5 was rarely expressed in normal mucosal epithelial cells and Lgr5^+^ cells were mainly concentrated at the bottom of the gastric mucosa gland **(A)**. IHC staining on GC tissues shown that Lgr5^+^ cells in GC exhibit four grades of Lgr5 expression intensity: negative **(B)**, weak positive **(C)**, positive **(D)**, and strong positive **(E)**. Kaplan-Meier survival analysis in GC patients (*n* = 100) according to the Lgr5 expression status **(F)**. High Lgr5 expression patients showed significantly shorter 5-year OS than patients with low Lgr5 expression in their tumors by log-rank test (*P* = 0.0057). Cum, cumulative; GC, gastric cancer; IHC, immunohistochemistry; HE, hematoxylin-eosin staining; ISO, negative control; Lrg5, leucine-rich repeat containing G protein-coupled receptor 5; OS, overall survival.

In our study, serial sections with H&E stain and IHC were performed to evaluate Lgr5 expression and the location of Lgr5^+^ cells. As shown in results, Lgr5 protein was rarely expressed in normal mucosal epithelial cells and a small amount of Lgr5^+^ cells were mainly concentrated at the bottom of the gastric mucosa gland (Figure 1A). The results of IHC shown that almost all the tumor cells were Lgr5 absolutely positive or absolutely negative. So the percentage of positive cells was categorized as two groups: 0 (0%) or 4 (100%). Therefore, the final score of Lgr5 expression depended on the intensity of staining which was assigned to 4 grades: negative, weak positive, positive and strong positive (Figures 1B–E). Patients with negative and weakly positive Lgr5 expression were assigned to the low Lgr5 expression group, and patients with positive and strongly positive Lgr5 expression were assigned to the high Lgr5 expression group. In our study, patients in the high Lgr5 expression group had a poorer 5-year OS than those in the low Lgr5 expression group, as analyzed by Kaplan-Meier survival curves and log-rank test (*P* = 0.0057; Figure 1F).

### High Lgr5 Expression Correlated With Th2 Cytokine Shift in Tumor Immune Microenvironment

**Figure 2:**
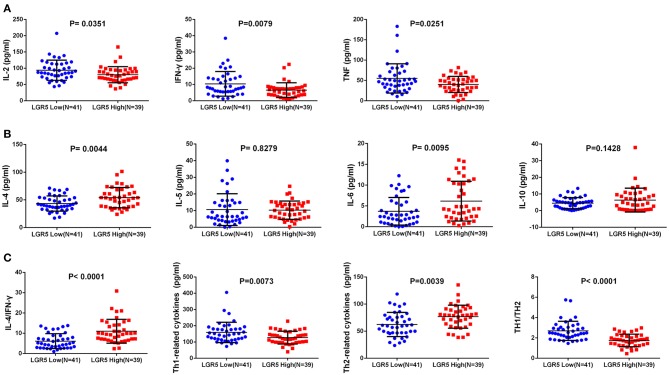
Th1/Th2 cytokine expression in frozen GC tissue samples detected by ELISA. **(A)** Th1 (pro-inflammatory) cytokines including IL-2, TNF-α, and IFN-γ. **(B)** Th2 (anti-inflammatory) cytokines including IL-4, IL-5, IL-6, and IL-10. **(C)** Total Th1 cytokines, total Th2 cytokines, IL-4/IFN-γ cytokines, total h1 cytokines/ total Th2 cytokines ratio. All the cytokines were detected in homogenate protein of fresh tissue (1μg/μl). Abbreviations: ELISA, enzyme-linked immunosorbant assay; GC, gastric cancer; IFN-γ, interferon gamma; IL, interleukin; Lrg5, leucine-rich repeat containing G protein-coupled receptor 5; TNF-α, tumor necrosis factor alpha.

The expression of Th1 cytokines (IL-2, TNF, IFN-γ) and Th2 cytokines (IL-4, IL-5, IL-6, IL-10) were examined in frozen tumor tissues from 80 GC patients by ELISA. Compared with concentrations in the low Lgr5 expression group, IL-4 and IL-6 concentrations were higher in the high Lgr5 expression group of patients (*P* = 0.0044 and *P* = 0.0095, respectively; 54.11 ± 18.45 pg/mL vs. 43.49 ± 13.69 pg/mL; 6.14 ± 4.79 pg/ml vs. 3.70 ± 3.31 pg/ml, respectively; Figure 2B). Contrary to these data, the concentration of IL-2, TNF-α, and IFN-γ in the high Lgr5 expression group were lower compared to the low Lgr5 expression group of patients (*P* = 0.0351; *P* = 0.0251 and *P* = 0.0079, respectively; 80.31 ± 24.73 pg/mL vs. 93.83 ± 31.11 pg/mL; 40.07 ± 19.80 pg/ml vs. 55.02 ± 36.00 pg/ml, 6.58 ± 4.52 pg/ml vs. 10.41±7.57 pg/ml, respectively; Figure 2A). IL-10 and IL-5 expression in high and low Lgr5 expression group did not differ significantly (*P* = 0.1428 and *P* = 0.8279, respectively; 6.33 ± 7.17 pg/ml vs. 4.49 ± 3.31 pg/ml; 10.25 ± 5.50 pg/ml vs. 10.63 ± 9.51 pg/ml, respectively; Figure 2B). Furthermore, expression of total Th2 cytokines (*P* = 0.0039, 76.83 ± 21.23 pg/ml vs. 62.32 ± 22.35 pg/ml; Figure 2C) and the IL-4/IFN-γ ratio (*P* < 0.0001, 11.02 ± 5.86 vs. 6.13 ± 3.66; Figure 2C) in the high Lgr5 expression group were much higher compared to the low Lgr5 expression group. In contrast, both total Th1 cytokine expression (*P* = 0.0073, 126.96 ± 39.33 pg/ml vs. 159.25 ± 62.31 pg/ml; Figure 2C) and the ratio of total Th1 cytokines to total Th2 cytokines (*P* < 0.0001, 1.75 ± 0.62 vs. 2.70 ± 0.95; Figure 2C) were lower in the high Lgr5 expression group.

### Intratumor Tregs Correlated With the Immunosuppressive Microenvironment and Poor Prognosis of GC Patients

**Figure 3:**
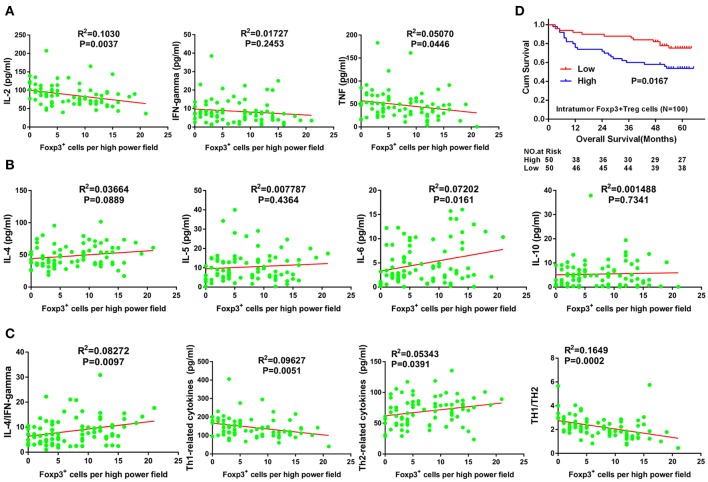
Correlation between Tregs number and Th1/Th2 cytokine expression, and its prognostic significance in GC. **(A)** Th1 (pro-inflammatory) cytokines including IL-2, TNF-α, and IFN-γ. **(B)** Th2 (anti-inflammatory) cytokines including IL-4, IL-5, IL-6, and IL-10. **(C)** Total Th1 cytokines, total Th2 cytokines, IL-4/IFN-γ ratio and total Th1 cytokines/total Th2 cytokines ratio. Cum, cumulative; GC, gastric cancer; IFN-γ, interferon gamma; IL, interleukin; TNF-α, tumor necrosis factor alpha.

In tumor tissue, the number of FoxP3^+^ Tregs was positively correlated with IL-6 expression (Figure 3B) and negatively correlated with IL-2 and TNF-α expression (Figure 3A) (*R*^2^ = 0.0720, *P* = 0.0161; *R*^2^ = 0.1030, *P* = 0.0037; and *R*^2^ = 0.0507, *P* = 0.0446, respectively). In addition, the number of FoxP3^+^ Tregs did not correlate with levels of IFN-γ, IL-4, IL-5 or IL-10 levels (*R*^2^ = 0.0173, *P* = 0.2453; *R*^2^ = 0.0366, *P* = 0.0889; *R*^2^ = 0.0078, *P* = 0.4364; and *R*^2^ = 0.0015, *P* = 0.7341, respectively; Figures 3A,B). Furthermore, the number of FoxP3^+^ Tregs positively correlated with the IL-4/IFN-γ ratio and total Th2 cytokines (*R*^2^ = 0.0827, *P* = 0.0097, and *R*^2^ = 0.0534, *P* = 0.0391, respectively; Figure 3C). By contrast, the number of FoxP3^+^ Tregs negatively correlated with total Th1 cytokines and the ratio of total Th1 cytokines to total Th2 cytokines in tumor tissues (*R*^2^ = 0.0963, *P* = 0.0051, and *R*^2^ = 0.1649, *P* = 0.0002, respectively; Figure 3C). With univariate analysis and the median value of intratumor FoxP3^+^ Tregs used as a cut-off, patients with a higher number of intratumor infiltrating FoxP3^+^ Tregs had a poorer OS than those with lower intratumor FoxP3^+^ Treg counts (*P* = 0.0167, Figure 3D).

### High Lgr5 Expression in Tumor Cells Positively Correlated With Number of Intratumor FoxP3+Tregs and TGF-β1 Expression

**Figure 4:**
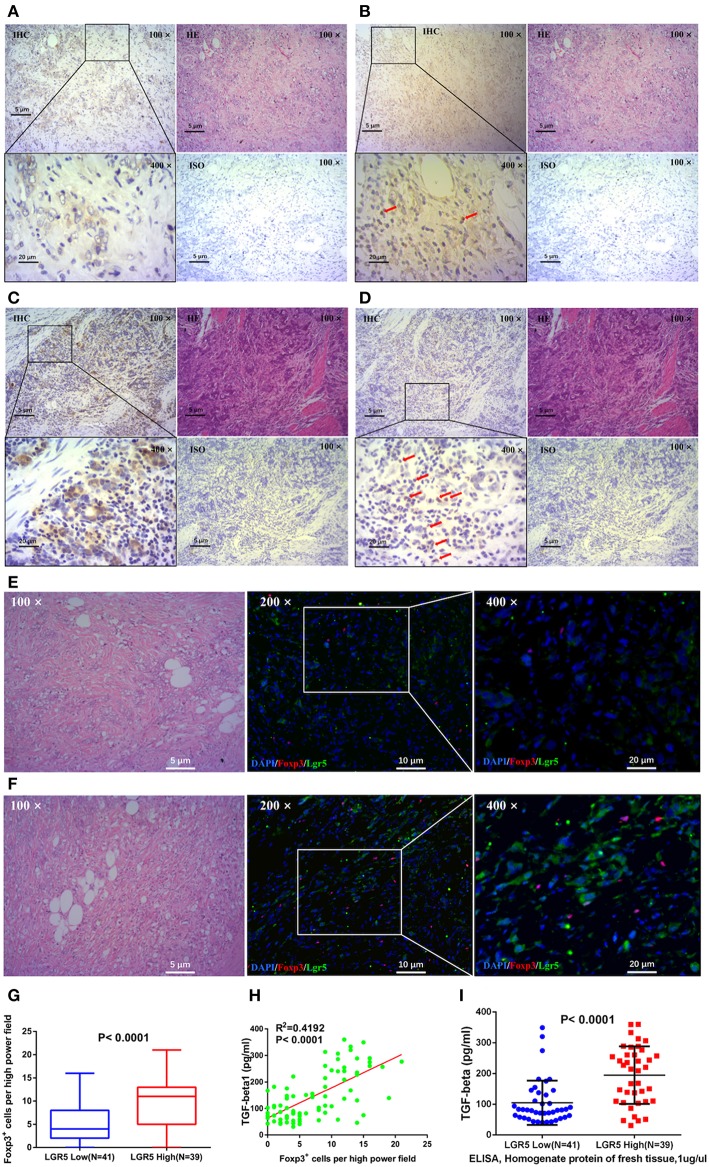
Correlation between Lgr5 expression, number of Tregs, and TGF-β1 expression in tumor tissues. IHC staining for Lgr5 **(A,C)** and FoxP3 **(B,D)** in GC tissue samples (brown, shown at 100× and 400× magnification). The red arrow indicates intratumor FoxP3^+^ Tregs detected in tumor tissues. IF analysis of GC tissues stained for Lgr5 (green) and FoxP3^+^ (red), and counterstained with DAPI (blue). There were less FoxP3^+^ Tregs in gastric cancer tissues with low Lgr5 expression **(E)**, while correspondingly, there were more FoxP3^+^ Tregs in cases with high Lgr5 expression **(F)**. Based on the results of IHC, statistical result of significant difference was obtained (*P* < 0.0001; **G**). **(H)** Correlation between number of Tregs and level of TGF-β1 in tumor tissues. **(I)** Gastric tumor tissue with higher Lgr5 expression also had significantly higher TGF-β1 levels (*P* < 0.0001). GC, gastric cancer; IF, immunofluorescence; IHC, immunohistochemistry; HE, hematoxylin-eosin staining; DAPI, 4′,6-diamidino-2-phenylindole; ISO, negative control; Lrg5, leucine-rich repeat containing G protein-coupled receptor 5; TGF-β1, transforming growth factor beta 1.

The relationship between the number of intratumor FoxP3^+^ Tregs and Lgr5 expression in tumor tissues was analyzed by IHC and IF. Serial sections of GC tissue samples were used for IHC staining of Lgr5 and FoxP3. These results demonstrated that there were more intratumor FoxP3^+^ Tregs in tumor tissues with higher Lgr5 expression (Figures 4A–D) and this phenomenon was more obvious using double immunofluorescence (Figures 4E,F). Further statistical analysis based on IHC suggested a significant positive correlation between FoxP3^+^ Tregs number and Lgr5 expression in tumor tissues (*P* < 0.0001; 9.77 ± 5.23 vs. 5.20 ± 4.50; Figure 4G). In addition, expression of the main cytokine secreted by Tregs, TGF-β, was also examined in frozen tumor tissues. A significant positive correlation between the intratumor FoxP3^+^ Tregs and the level of TGF-β1 was observed in tumor tissue of GC patients (*R*^2^ = 0.4192, *P* < 0.0001; Figure 4H). Furthermore, in the fresh tumor tissues, the level of TGF-β1 in the high Lgr5 expression group were much higher compared with the low Lgr5 expression group (*P* < 0.0001; 194.77 ± 93.74 pg/ml vs. 105.13 ± 71.90 pg/ml; Figure 4I).

### Tregs Promoted Lgr5 Expression in GC Cells *in vitro* via TGF-β1

**Figure 5:**
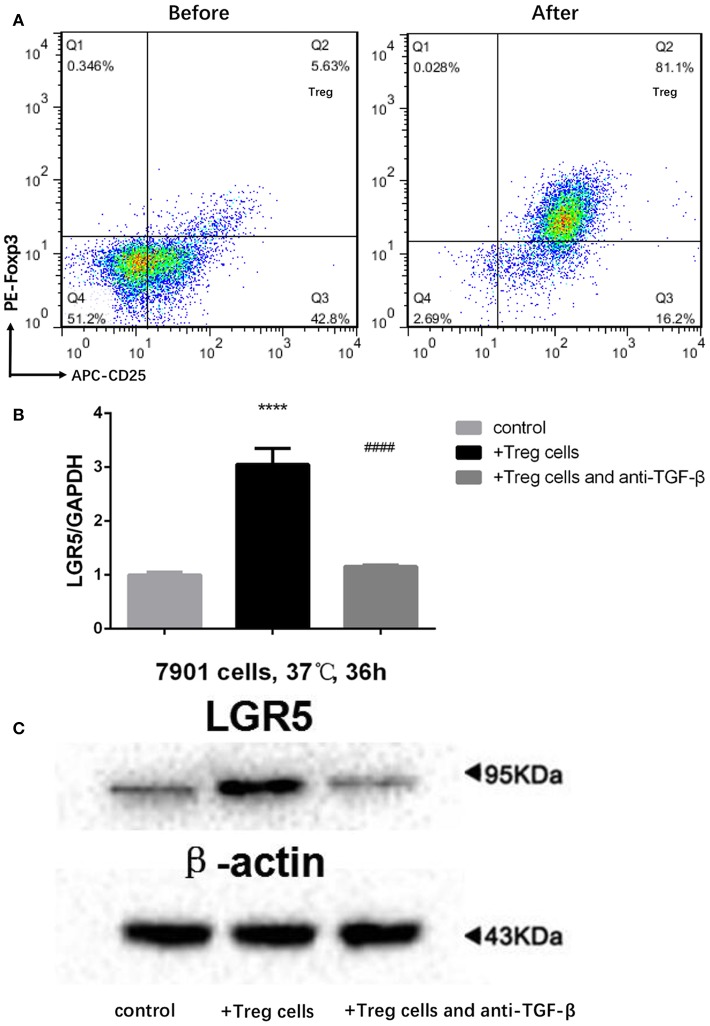
Treg-derived TGF-β1 as the main factor inducing high Lgr5 expression in GC cells. **(A)** Flow cytometric CD4-gated analysis of CD25^+^FoxP3^+^ cells was carried out in GC patient samples before and after MACS. **(B,C)** Tregs induce high Lgr5 expression in GC cells. GC cells were treated with Tregs (1 × 104 cells/mL) with and without TGF-β1 neutralizing antibody (1 μg/ml) for 36 h. Lgr5 expression was detected by western blot and qRT-PCR. mRNA expression was normalized to GAPDH. ****P < 0.0001 between Treg-treated SGC-7901 cells and control group. ^*####*^*P* < 0.0001 between Treg-treated SGC-7901 cells with or without TGF-β1 neutralizing antibody (1 μg/mL). GAPDH, glyceraldehyde 3-phosphate dehydrogenase; GC, gastric cancer; Lrg5, leucine-rich repeat containing G protein-coupled receptor 5; MACS, magnetic-activated cell sorting; qRT-PCR, quantitative revere transcription polymerase chain reaction; TGF-β1, transforming growth factor beta 1.

Tregs in the peripheral blood of GC patients were separated by MACS. Following MACS, the fraction of CD4^+^CD25^+^FoxP3^+^ Tregs increased from 5.63 to 81.1% (Figure 5A). Furthermore, Lgr5 expression in several GC cell lines (AGS, SGC-7901, MGC-803, MKN45, BGC-823, and KATO III) was examined by flow cytometry and western blot. The results demonstrate that Lgr5 expression varied by cell line, and the cells were then divided into Lgr5^+^ and Lgr5^−^ cell subtypes by flow cytometry (Supplementary Figure 1A). The SGC-7901 cell line (26.5% Lgr5^+^ cells), which had the highest Lgr5 expression among the examined GC cell lines, was chosen to co-culture with Tregs isolated from the peripheral blood of patients with GC (Supplementary Figure 1B). Lgr5 expression in SGC-7901 cells was enhanced by Tregs, following the incubation of tumor cells with 1 × 10^4^ Tregs/mL Tregs for 36 h. Next, TGF-β1 neutralizing antibody (1 μg/mL) was used to neutralize the TGF-β1 secreted by Tregs. After immunodepletion of TGF-β1, Tregs failed to promoted Lgr5 expression in tumor cells, which indicated that Treg-derived TGF-β1 was the main factor that induced Lgr5 expression (Figures 5B,C).

### TGF-β1 Regulated the Overexpression of Lgr5 and β-Catenin in GC Cells via TGF-β1 Signaling Pathway

**Figure 6:**
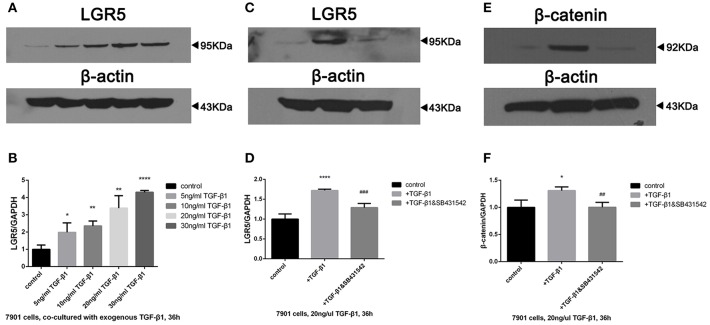
TGF-β1 regulated the overexpression of Lgr5 and β-catenin in GC cells via TGF-β1 signaling pathway. **(A,B)** Lgr5 expression in GC cells exposed to 0, 5, 10, 20, and 30 ng/ml TGF-beta1 for 36 h determined by Western blot **(A)** and Q-PCR **(B)**. **(C–F)** GC cells were treated with 20 ng/ml TGF-beta1 in the presence of 0, 10 uM SB431542 for 36 h, Lgr5 expression **(C,D)** and beta-catenin **(E,F)** in GC cells was detected by Western blot **(C,E)** and Q-PCR **(D,F)**. The mRNA level was normalized to that of GAPDH in the same cell extracts. *P < 0.05, **P < 0.01, ****P < 0.0001 between GC cells treated with different concentration of TGF-beta1 and control group. ^*##*^*P* < 0.01, ^*###*^*P* < 0.001 between TGF-beta1-treated GC cells with or without 10uM SB435421. GAPDH, glyceraldehyde 3-phosphate dehydrogenase; GC, gastric cancer; Lrg5, leucine-rich repeat containing G protein-coupled receptor 5; MACS, magnetic-activated cell sorting; qRT-PCR, quantitative revere transcription polymerase chain reaction; TGF-β1, transforming growth factor beta 1.

In order to examine the role of TGF-β1 in gastric cancer, SGC-7901 cells were exposed to various concentrations of exogenous TGF-β1 ranging from 5 to 30 ng/mL for 36 h, and Lgr5 expression was shown to be concentration-dependent (Figures 6A,B). Next, SB431542, a selective antagonist of TGF-β1/ALK5/Smad2 signaling, was added to SGC-7091 cells treated with various TGF-β1 concentrations. As a result, Lgr5 induction was partially inhibited in cells exposed to exogenous TGF-β1 (20 ng/μL) and SB431542 (10 μM; Figures 6C,D). In addition, when SGC-7901 cells were treated with TGF-β1, the expression of β-catenin (canonical component of Wnt signaling pathway) was also increased. Moreover, this up-regulation was partially inhibited by SB431542 (Figures 6E,F).

### Lgr5 Expression in GC Tissue Was an Independent Prognostic Factor

In total, 100 GC patients (68 males and 32 females) were recruited for this study between February 2009 and March 2010. The clinicopathological characteristics of these patients are presented in Table 1. The age of enrolled GC patients ranged from 33 to 85 years (median 60 ± 11 years). Of the 100 patients, 35 (35%) died during the observation period. The median follow-up time was 56 months, ranging from 2 to 66 months. The OS rates were 84% at 1 year, 74% at 3 years, and 65% at 5 years.

**Table 1:** Univariate and Multivariate analysis of factors associated with overall survival in 100 gastric cancer patients.

	**Univariate^b^**	**Multivariate**	
**Variables**	***P-value***	**HR(95% CI)**	***P*-value**
Gender (Male vs. Female)	0.6035		NA
Age (years)(>60 vs. ≤ 60)	0.1015		NA
Tumor size (>5 cm vs. ≤ 5 cm)	0.1457		NS
Degree of differentiation (Poorly vs. well and moderately)	0.1438		NS
TNM stage^a^ (III vs.Tis+I+II)	0.0002	0.1888 (0.0816–0.4369)	<0.0001
Lgr5 expression (High vs. Low)	0.0057	0.2888 (0.1372–0.6078)	0.0010
Number of Foxp3+ Tregs (High vs. Low)	0.0167		

*HR, hazard ratio; CI, confidence interval; NA, not applicable; NS, not significant*.

a *7th Edition of American Joint of Committee On Cancer*.

b *Univariate analysis were performed by the Kaplan-Meier analysis model and log-rank test*.

*^c^ Multivariate analysis were performed by Cox proportional hazards models with the backward likelihood stepwise procedures*.

The chi-squared test or Fisher's exact test were used to evaluate the correlation between Lgr5 expression, intratumor FoxP3^+^ Tregs, and clinicopathological features. However, neither Lgr5 expression nor intratumor FoxP3^+^ Tregs correlated with any clinicopathological characteristics, including tumor size, degree of differentiation and TNM classification (Supplementary Table 1). Furthermore, with univariate analysis, high expression of Lgr5, high number of intratumor infiltrating FoxP3^+^ Tregs, and advanced TNM stage were all predictors of poor prognosis (Table 1). Finally, tumor size, degree of differentiation, TNM stage, Lgr5 expression level, and intratumor FoxP3^+^ Tregs were included in a multivariate Cox proportional hazards analysis. TNM stage (HR = 0.1888; 95% CI: 0.0816–0.4369; *P* < 0.0001) and Lgr5 expression level (HR = 0.2888; 95% CI: 0.1372–0.6078; *P* = 0.001) were identified as independent prognostic factors.

## Discussion

Lgr5, a downstream target in Wnt/β-catenin signaling, has also been established as a stem cell marker in the stomach and intestine (30). Moreover, Lgr5 is also a putative marker of gastrointestinal cancer stem cells (6, 29). Indeed, it has been shown that Lgr5^+^ cells are the cells of origin in mouse models of gastrointestinal cancer (1, 29). Furthermore, in human gastric and colorectal cancer tissues, Lgr5^+^ cancer cells have been shown to exhibit properties of cancer stem cells (CSCs) or cancer-initiating cells (9, 33). Moreover, Lgr5 is highly expressed in different human gastrointestinal tumors originating from colon, liver, pancreas, and stomach when compared to its expression in the corresponding normal tissues (2).

In this study, Lgr5 expression was examined using IHC and the data indicated that Lgr5 was widely expressed in GC tissue. Contrary to this, Lgr5 was rarely expressed in normal gastric mucosa, which was consistent with the reports from other authors (2). Furthermore, we divided GC patients into high or low Lgr5 expression groups according to the intensity of the IHC stain and the number of Lgr5 positive cells. As a result, in our study GC-associated Lgr5 overexpression was found to be significantly associated with poor OS of GC patients. High expression of Lgr5 could therefore be used as a potential marker of poor prognosis for patients with GC. Nevertheless, the molecular and cellular mechanisms of the poor prognosis associated with high Lgr5 expression in GC patients needs to be elucidated.

Lgr5 is a Wnt/β-catenin signaling response gene and it has been previously demonstrated that β-catenin promotes intratumoral Treg responses in mouse models (34). Tregs are able to exert their tumor-specific immunosuppressive responses by creating an immunosuppressive tumor microenvironment, thus promoting cancer progression that results in poor patient prognosis, in many types of cancer including GC, colorectal cancer, and other malignancies (22, 23, 35–37). We and other authors have demonstrated that patients with GC have an increased prevalence of both circulating and tumor-infiltrating CD4^+^CD25^+^FoxP3^+^ Tregs in comparison to the healthy population (19, 20, 22). Moreover, tumor-infiltrated FoxP3^+^ Tregs are significantly associated with poor OS of GC patients (38–40). In the current study, 100 GC patients were divided into high or low tumor-infiltrating Treg groups according to the median number of intratumoral FoxP3^+^ Tregs as the cut-off value. As a result, we have observed that a high number of tumor-infiltrating Tregs was also significantly associated with poor OS in GC patients. Taken together, our study has shown that the high expression of Lgr5 and high number of tumor-infiltrating Tregs are both associated with poor OS in patients with GC.

Furthermore, our results of univariate analysis indicated that TNM stage (Tis+I+II vs. III), Lgr5 expression (low vs. high), and number of tumor infiltrating Tregs (low vs. high) were associated with OS in GC patients. Moreover, multivariate analysis indicated that the TNM stage and Lgr5 expression were independent risk factors for OS. Collectively, univariate and multivariate analysis results indicate that high Lgr5 expression in association with a high number of tumor-infiltrating Tregs promotes cancer progression and poor prognosis in GC. The underlying mechanisms behind their co-contribution to the prognosis of GC have yet to be examined in future studies.

In the tumor microenvironment, Tregs and other immune cells produce a complex mixture of soluble cytokines, chemokines and growth factors. Previous studies have shown that a remarkable increase in Th2 cytokines (anti-inflammatory cytokines: IL-4, IL-5, IL-6, and IL-10), and simultaneous decrease of Th1 cytokines (pro-inflammatory cytokines: TNF-α, IFN-γ, and IL-2) are often present as a hallmark feature of the tumor microenvironment, including tumors such as hepatocellular carcinoma, non-small-cell lung carcinoma, and other types of cancer (40–44). Indeed, it has been suggested that a Th2 shift in the tumor microenvironment contributes to tumor relapse, metastasis, and poor prognosis. In this study, Th1 and Th2 cytokines were examined in fresh-frozen human GC tissues by ELISA. Based on our data, a shift toward the production of Th2 cytokines occurs in the tumor microenvironment of GC patients with high expression of Lgr5. Furthermore, this result suggests that an increase in Lgr5 expression is associated with a tumor-suppressive immune microenvironment in GC patients.

To further explore the tumor microenvironment in GC, we have focused on intratumor FoxP3^+^ Tregs, in order to examine whether Tregs participate in the regulation of the tumor milieu. In the present study, the immunosupresive microenvironment of gastric tumor tissue was positively correlated with the number of intratumor FoxP3^+^ Tregs, which indicates that intratumor FoxP3^+^ Tregs may play an important role in the promotion of these immunosupresive conditions. The higher Lgr5 levels in GC tumors with a high number of intratumor FoxP3^+^ Tregs observed in our study indicated that intratumor FoxP3^+^ Tregs may influence Lgr5 expression in tumor tissue. It has been shown previously that Tregs exert their immunomodulatory properties through high and stable expression of TGF-β1 (45, 46). It has also been established that Tregs are one of the major sources of TGF-β1. According to previous reports, higher number of Tregs in non-small-cell lung carcinoma and ovarian cancer were positively correlated with high levels of TGF-β1 secretion (47). In the current study, we demonstrated a high correlation between the number of Tregs and TGF-β1 expression. Furthermore, we have shown that gastric tumors with higher expression of Lgr5 also exhibit higher levels of TGF-β1 expression. Moreover, in the co-culture of Tregs with GC cells, Tregs seemed to promote Lgr5 expression via TGF-β1.

The TGF-β1 signaling pathway participates in the regulation of many pivotal biological processes, including cell proliferation, differentiation, migration, and apoptosis, and is one of the most frequently altered pathways in human cancers (48). The TGF-β1 signaling pathway exerts its biological activity through the specific intracellular mediators known as Smad proteins, which in turn interact with multiple other signaling pathways (49). TGF-β serves as a tumor-ressive cytokine in the normal intestinal epithelium, due to its involvement in the inhibition of cell proliferation and induction of apoptosis. However, colorectal tumors can escape from the tumor-suppressive effects of TGF-β (50). Contrary to its previously described role, TGF-β1 has been reported to act as tumor promoter in advanced CRC and its expression was shown to be significantly increased (25). Indeed many studies thus far confirm that disruption of both the TGF-β1 and Wnt signaling pathways synergistically drive CRC tumorigenesis, likely in stem cells, and affect CRC progression *in vivo* (27, 28). Wnt pathway activation contributes to carcinogenesis in GC, and Lgr5 is a downstream target of this pathway (30). In addition, up-regulation of Lgr5 in various tumor types with increased Wnt signaling has also been reported (3–5, 8). Therefore, we hypothesized that the correlation between Lgr5 and TGF-β1 expression may be due to the interaction between TGF-β1 and Wnt signaling pathways. In the current study, Lgr5 expression in SGC-7901 cells was enhanced by exogenous TGF-β1 treatment, and this effect was receded after the TGF-β1/ALK5/Smad2 inhibitor was applied. Several other studies have reported that Lgr5 is highly expressed in tumor tissues with nuclear accumulation of β-catenin (3), which is crucial for the activation of the Wnt signaling pathway (51). Moreover, it has been reported that Lgr5 expression is down-regulated in CRC when Wnt signaling is suppressed (52). In this study, β-catenin expression followed the same trend as Lgr5 expression, which suggested that exogenous TGF-β1 may could activate the Wnt signaling pathway through the TGF-β1/ALK5/Smad2 signaling pathway, which lead to the up-regulation of Lgr5 expression in GC cells (Supplementary Figure 2). This assumption will be validated in our next study.

## Conclusion

In this study we have outlined an immunosuppressive environment of gastric tumors with high expression of Lgr5, and the complexity of the anti-inflammatory cytokine network associated with it. Our findings support the idea that Tregs promote the high expression of Lgr5 in GC cells via TGF-β1 and TGF-β1 signaling pathway, which may involve activation of the Wnt signaling pathway. Finally, we have for the first time reported that high expression of Lgr5 promoted by Tregs confer poor prognosis in GC patients. Therefore, these newly established network interactions in gastric cancer might prove useful in providing new potential targets for antitumor therapy.

## Data Availability

The raw data supporting the conclusions of this manuscript will be made available by the authors, without undue reservation, to any qualified researcher.

## Ethics Statement

This study was carried out in accordance with the recommendations of the ethical committee of the First Affiliated Hospital, Medical College, Zhejiang University (Hangzhou; Ethical number: 2017380) with written informed consent from all subjects. All subjects gave written informed consent in accordance with the Declaration of Helsinki. The protocol was approved by the recommendations of the ethical committee of the First Affiliated Hospital, Medical College, Zhejiang University.

## Author Contributions

J-RY was mainly responsible for the design of the experiment. X-SL and X-KL were mainly responsible for specific experiments. YM and SA were primarily responsible for data analysis. C-XY and H-LJ were mainly responsible for sample collection. HY, CC, and C-ZL were mainly responsible for clinical data collation and patient follow-up.

### Conflict of Interest Statement

The authors declare that the research was conducted in the absence of any commercial or financial relationships that could be construed as a potential conflict of interest.
