# Supplementary material for: Regulatory T Cells Promote Overexpression of Lgr5 on Gastric Cancer Cells via TGF-beta1 and Confer Poor Prognosis in Gastric Cancer
Source: Front Immunol. 2019 Jul 30;10:1741. doi: 10.3389/fimmu.2019.01741 (PMC6682668; doi:10.3389/fimmu.2019.01741)
Supplement: Supplementary file 1 [file Data_Sheet_1.docx]

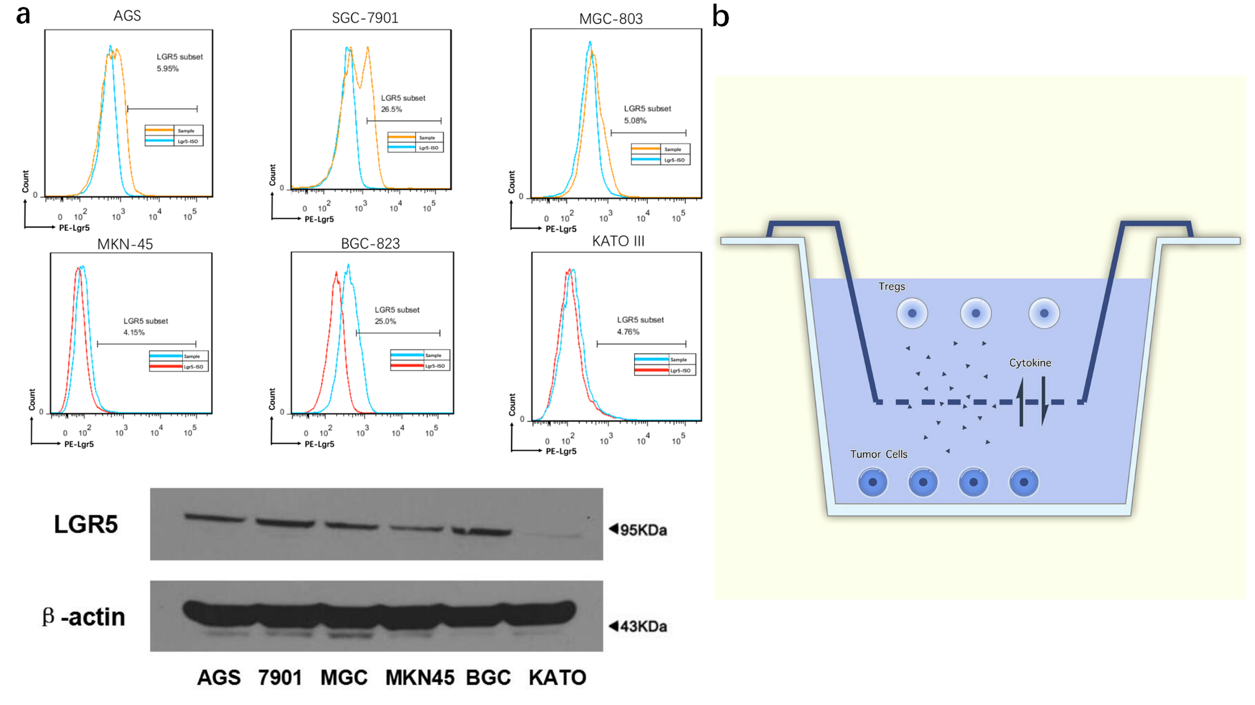


**Suppl. Fig. 1.** GC cell line selection and treatment. a. Lgr5 expression in different GC cell lines (AGS, SGC-7901, MGC-803, MKN45, BGC-823 and KATO III) determined by flow cytometry and western blot. b. Model of co-culture between Tregs and GC cells used in this study. Abbreviations: GC, gastric cancer; Lrg5, leucine-rich repeat containing G protein-coupled receptor 5.


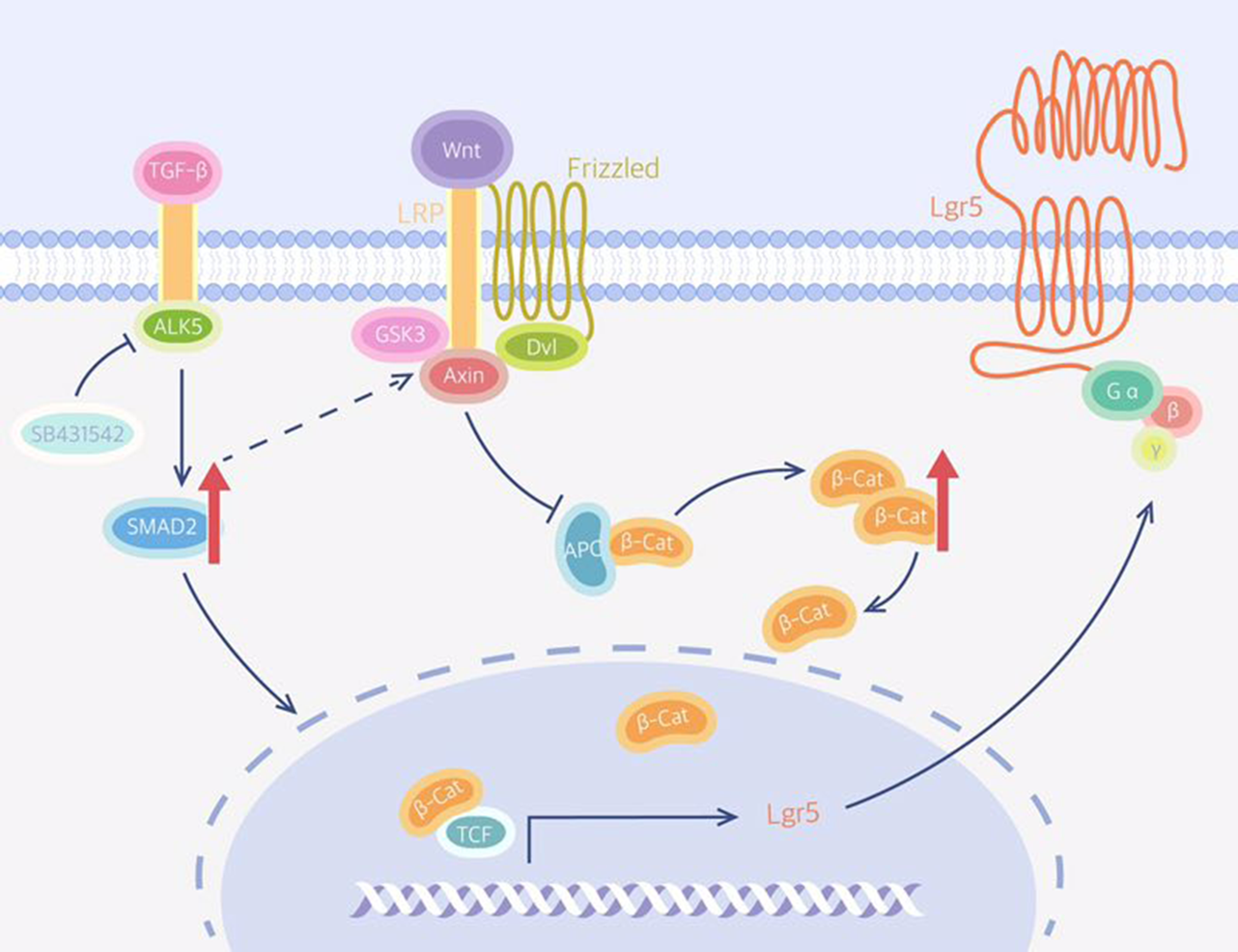


**Suppl. Fig. 2.** Putative model of TGF-β signaling pathway activation leading to Smad2 upregulation and activation of the canonical Wnt signaling pathway. Recruitment of Axin results in the inactivation of APC destruction complex and subsequent stabilization of β-catenin. β-catenin in turn translocates to the nucleus to bind TCF transcription factors, which leads to the up-regulation of the Wnt target gene Lgr5. Lgr5 encodes a seven-transmembrane protein with a large extracellular domain for ligand binding and a short cytoplasmic tail for coupling to G proteins. Abbreviations: APC, adenomatous polyposis coli; Lrg5, leucine-rich repeat containing G protein-coupled receptor 5; TCF, T cell factor, TGF-β1, transforming growth factor beta 1.
