# Supplementary material for: Regulatory T Cells Promote Overexpression of Lgr5 on Gastric Cancer Cells via TGF-beta1 and Confer Poor Prognosis in Gastric Cancer
Source: Front Immunol. 2019 Jul 30;10:1741. doi: 10.3389/fimmu.2019.01741 (PMC6682668; doi:10.3389/fimmu.2019.01741)
Supplement: Supplementary file 2 [file Table_1.doc]

**Supplementary Table. Association between clinicopathological characteristics and Lgr5 and Tregs.**

|  | **Lgr5 expression** | | |  | **Number of Foxp3^+^ Tregs** | | |
| --- | --- | --- | --- | --- | --- | --- | --- |
| **Variables (No.)** | **Low** | **High** | **P-value** |  | **Low** | **High** | **P-value** |
| Gender  Male (68)  Female (32) | 34  13 | 34  19 | 0.3809 |  | 37  13 | 31  19 | 0.1984 |
| Age  *≤*60 (53)  >60 (47) | 26  21 | 27  26 | 0.6617 |  | 24  26 | 29  21 | 0.3164 |
| Tumor size  ≤5cm (82)  >5cm (18) | 40  7 | 42  11 | 0.4464 |  | 40  10 | 42  8 | 0.6027 |
| Degree of differentiation  Well and moderately (25)  Poorly (75) | 11  36 | 14  39 | 0.7286 |  | 18  32 | 7  43 | 0.0111 |
| T stage^a^  Tis+T1+T2 (35)  T4 (65) | 14  33 | 21  32 | 0.3034 |  | 16  34 | 19  31 | 0.5294 |
| Lymph node metastasis^a^  N0 (30)  N1+N2+N3(70) | 12  35 | 18  35 | 0.3585 |  | 18  32 | 12  38 | 0.1904 |
| TNM stage^a^  Tis + I + II (46)  III (54) | 19  28 | 27  26 | 0.2922 |  | 23  27 | 23  27 | 1.0000 |

^a^7^th^ Edition of American Joint of Committee On Cancer.
